# Potential Role of A_2B_ Adenosine Receptors on Proliferation/Migration of Fetal Endothelium Derived from Preeclamptic Pregnancies

**DOI:** 10.1155/2014/274507

**Published:** 2014-04-28

**Authors:** Jesenia Acurio, Felipe Troncoso, Patricio Bertoglia, Carlos Salomon, Claudio Aguayo, Luis Sobrevia, Carlos Escudero

**Affiliations:** ^1^Vascular Physiology Laboratory, Group of Investigation in Tumor Angiogenesis (GIANT), Group of Research and Innovation in Vascular Health (GRIVAS Health), Department of Basic Sciences, Faculty of Sciences, Universidad del Bío-Bío, Chillán, Chile; ^2^Obstetrics and Gynecology Department, Herminda Martin Clinical Hospital, Chillan, Chile; ^3^University of Queensland Centre for Clinical Research (UQCCR), Faculty of Medicine and Biomedical Sciences, University of Queensland, Herston, QLD 4006, Australia; ^4^Department of Clinical Biochemistry and Immunology, Faculty of Pharmacy, University of Concepción, Chile; ^5^Cellular and Molecular Physiology Laboratory (CMPL), Division of Obstetrics and Gynecology, Faculty of Medicine, School of Medicine, Pontificia Universidad Católica de Chile, Santiago, Chile

## Abstract

To investigate the functionality of A_2B_ adenosine receptor (A_2B_AR) and the nitric oxide (NO) and vascular endothelial growth factor (VEGF) signaling pathway in the endothelial cell proliferation/migration during preeclampsia, we used human umbilical vein endothelial cells (HUVECs) isolated from normal pregnancies (*n* = 15) or pregnancies with preeclampsia (*n* = 15). Experiments were performed in presence or absence of the nonselective adenosine receptor agonist NECA, the A_2B_AR selective antagonist MRS-1754, and the nitric oxide synthase (NOS) inhibitor L-NAME. Results indicated that cells from preeclampsia exhibited a significant higher protein level of A_2B_AR and logEC_50_ for NECA-mediated proliferation than normotensive pregnancies. The stimulatory effect of NECA (10 **μ**M, 24 h) on cell proliferation was prevented by MRS-1754 (5 nM) coincubation only in cells from normotensive pregnancies. Nevertheless, L-NAME (100 **μ**M, 24 h) reduced the NECA-induced cell proliferation/migration in HUVEC from normal pregnancy; however in preeclampsia only NECA-induced cell proliferation was reduced by L-NAME. Moreover, NECA increased protein nitration and abundance of VEGF in cells from normal pregnancy and effect prevented by MRS-1754 coincubation. Nevertheless, in preeclampsia NECA did not affect the protein level of VEGF. In conclusion HUVECs from preeclampsia exhibit elevated protein level of A_2B_AR and impairment of A_2B_AR-mediated NO/VEGF signaling pathway.

## 1. Introduction

Preeclampsia is the leading cause for maternal and neonatal morbidity and mortality worldwide [1]. The pathophysiology of the fetal complications in preeclampsia is still unclear, but it has been associated with placental alterations compatible with under perfusion [2, 3] and reduced placental blood flow [4], which in turn may limit the delivery of nutrients to the fetus. Since placental and proximal umbilical vessels lack innervation [5], the regulation of vascular blood flow depends on the synthesis and release of endothelial vasoactive molecules, such as nitric oxide (NO) and adenosine [6–8]. In this regard, several evidences suggest that preeclampsia is characterized by endothelial dysfunction in both maternal [9, 10] and fetoplacental circulation [11–14], which may impair not only vascular tone regulation but also angiogenesis.

Adenosine is a naturally occurring nucleoside, which controls several physiological processes including vascular tone regulation and angiogenesis [15, 16], via activation of G-protein-coupled adenosine receptors (AR) [16]. Four types of AR have been identified: A_1_AR, A_2A_AR, A_2B_AR, and A_3_AR [16, 17]. At the physiological nanomolar range, adenosine mainly activates A_1_AR, A_2A_AR, and A_3_AR, whereas A_2B_AR requires micromolar concentrations [18]. Nevertheless, exposure of any AR to agonists for shorter or longer times generally leads to the attenuation of the agonist response. In this regard, desensitization of A_2B_ receptor has been described in different cell lines (see details in [18]), but little is known regarding A_2B_AR desensitization during human diseases.

In the fetoplacental circulation from preeclampsia, elevated level of adenosine has been reported in umbilical vein (1.7 versus 0.5 *μ*mol/L, preeclampsia versus normal pregnancy, resp.) [19, 20] and in culture medium of human placental microvascular endothelial cells (hPMEC) (2.7 versus 0.6 *μ*mol/L, preeclampsia versus normal pregnancy, resp.) [7], suggesting that under this pathological condition all subtypes of adenosine receptor would be activated [18]. However, little is known regarding the potential role of adenosine receptors in the regulation of placental homeostasis during preeclamptic pregnancies. In particular, regarding A_2B_AR, high levels of this receptor have been reported in total placental homogenate [21], but no changes in hPMEC [7] derived from preeclampsia compared to normal pregnancy. Interestingly, elevated adenosine uptake seen in hPMEC from preeclampsia reverted by A_2A_AR/A_2B_AR inhibitors, suggest a tonic activation of adenosine transport by these receptors in preeclamptic pregnancies [7]. Nevertheless, other groups have reported that activation of A_2B_AR in HUVEC exposed to hypoxia [22] or not is associated with upregulation of proangiogenic factors such as vascular endothelial growth factor (VEGF) [23–26] and NO [27]. Stimulation of A_2B_AR increases proliferation of several cell types including porcine, rat endothelium [28], and human retinal endothelial cell [15]. Notwithstanding, in HUVEC from preeclampsia occurring before 34 weeks of gestation, we have described reduced cell proliferation associated with decreased activation of A_2A_AR/NO signaling pathway compared to controls [29]. Despite these evidences, it is unknown whether preeclampsia may affect the proangiogenic role of A_2B_AR in fetal endothelium.

We propose that HUVECs isolated from preeclamptic pregnancies exhibit reduced activation of A_2B_AR compared to normal pregnancy.

## 2. Patients and Methods

### 2.1. Reagents

Unless otherwise indicated, all reagents were purchased form Sigma-Aldrich (MO, USA): nonselective agonist for adenosine receptors, NECA, 5′-(N-ethylcarboxamido)adenosine; selective A_2B_AR antagonist, MRS-1754, 8-{4-{((4-cyanophenyl) carbamoylmethyl)oxy}phenyl}-1,3-di(n-propyl) xanthine hydrate; nonselective inhibitor of nitric oxide synthase, L-NAME, N_*ω*_-nitro-L-arginine methyl ester hydrochloride; antibodies: anti-A_2B_AR (Santa Cruz Biotechnology, CA, USA); antinitrotyrosine (Merck Millipore, MA, USA); anti-eNOS (Transduction Laboratories, NJ, USA), anti-VEGF (Cell Signaling, MA, USA), and anti-*β*-actin (Sigma Aldrich, MO, USA).

### 2.2. Patients

The Ethical Committee from the Universidad del Bío-Bío approved this cohort study and the informed consent was obtained from each participant. Pregnant women who attended to the Obstetrics and Gynecology Department of the Herminda Martin Clinical Hospital, Chillan, Chile, for their delivery were included in this study. Exclusion criteria included chronic hypertension, altered renal function, diabetes, chronic diseases, twin pregnancies, recurrent miscarriages, and abruption placenta. Women were classified into normal pregnancy (maternal blood pressure < 140/90 mmHg, absence of proteinuria, and no medical complications) and preeclampsia (new onset hypertension defined as blood pressure ≥ 140/90 mmHg, with at least 2 measurements 6 h apart, and proteinuria > 300 mg/24 hours after 20 weeks of gestation). The gestational age was defined as the period of time from the first day of the mother's last menstrual period and the delivery date, confirming this age by a first trimester ultrasound. Moreover, according to the standard protocol, all patients with preeclampsia received antihypertensive treatment and magnesium sulfate to prevent convulsions in case of severe preeclampsia. Diagnosis of small gestational age was performed when the newborn weight was <10th percentile to Chilean population [30].

### 2.3. HUVEC Culture

Endothelial cells were isolated from the human umbilical vein by digestion with collagenase (0.25 mg/mL) and then cultured (37°C, 5% CO_2_) in medium 199 (M199) as previously described [31]. All experiments were performed in duplicate, after overnight serum deprivation and in presence of adenosine deaminase (ADA 1 IU/mL) (Merck Millipore, Darmstadt, Germany). Cells were used in passage 2.

### 2.4. Cell Proliferation and Migration

Cell proliferation was analyzed after treatment (24 h) with NECA (10 *μ*M) and/or MRS-1754 (5 nM) or L-NAME (100 *μ*M) by using 3-(4,5-dimethylthiazol-2-yl)-5-(3-carboxymethoxyphenyl)-2-(4-sulfophenyl)-2H-tetrazolium (MTS) assay following the manufacture instruction (Promega, WI, USA) as described previously [29]. Moreover, in parallel experiments, cell migration was analyzed by transwell chambers assay as described elsewhere. In brief, HUVECs were trypsinized and seeded in the upper compartment of the transwell (Corning, NY, USA) at a density of 150 × 10^3^ cells/well in M199. The lower compartments were loaded with culture medium (control) or AR agonist and/or antagonist. After 24 hours, cells that had migrated to the bottom of the transwell membrane (8 *μ*m) were stained using hematoxylin (Winkler, Santiago, Chile). Membranes were observed at 40x magnification using a light microscope (Olympus, Tokyo, Japan) and 3 photos were taken from each preparation [29]. In addition, both cell proliferation and migration were measured in a concentration-response curve in presence of NECA.

### 2.5. Immunocytochemistry

The presence of A_2B_AR in HUVECs was evidenced by immunocytology following the manufacturers protocol (Vector Laboratories, CA, USA), as described previously [29]. Briefly, HUVECs were fixed in 4% paraformaldehyde prepared in phosphate buffer (PBS (mM): NaCl 13.7, KCl 2.7, Na_2_HPO_4_ 0.9, KH_2_PO_4_ 1.8, pH 7.4, 4°C) for 20 minutes. After blocking unspecific binding, cell preparations were incubated overnight with primary A_2B_AR antibody (1 : 200 v/v) followed by incubation with respective secondary antibody (1 : 500 v/v) diluted in PBS with 5% serum. Antigen-antibody reaction was further revealed by diaminobenzidine reaction (DAB). Analysis was blinded and performed using a bright field microscope (Olympus, Tokyo, Japan). For densitometric analysis three random pictures from each preparation were taken using a digital camera (Guangzhou Micro-shot Technology Co., Ltd, Guangdong, China). Estimation of the intensity of staining in the pictures was analyzed using ImageJ software (NIH, MD, USA) after light calibration and color deconvolution as previously described [32]. Values are expressed as the ratio of the area of positive brown stain divided by the total area of the reference field.

### 2.6. Western Blot

Cell, protein extracts (70 *μ*g) were separated by SDS-PAGE (10%) transferred to nitrocellulose membranes and probed with primary anti-A_2B_AR (1 : 2000 v/v), antinitrotyrosine (1 : 3000 v/v), anti-eNOS (1 : 2000 v/v), anti-VEGF (1 : 3000 v/v), and anti-*β*-actin (1 : 10000 v/v) antibodies diluted in PBS with 0.1% Tween (pH 7.4) and 5% milk. Then, a horseradish peroxidase-conjugated secondary antibody was used for visualization [29]. Secondary antibody was selected according to respective primary antibody. Dilution range of secondary antibody was 1 : 2000 to 1 : 150000 v/v in PBS/Tween buffer (pH 7.4) with 5% milk.

### 2.7. Nitrite Measurement

Nitrite levels were measured by the Griess reaction using a commercially available kit (Promega, WI, USA). In brief, confluent cells were incubated (30 min) in the presence or absence of NECA (10 *μ*M) and/or MRS-1754 (5 nM); and then, 100 *μ*L of M199 was collected for nitrite quantification in a spectrophotometer (Autobio, Zhengzhou, China) using a 540 nm filter [29].

### 2.8. Statistical Analysis

Comparisons between groups were performed by the Mann-Whitney test. We used *X* ^2^ test to analyze proportions. Values are mean ± S.E.M., where *n* indicates number of different cell cultures (in duplicate). *P* < 0.05 is considered statistically significant. The statistical software GraphPad Instat 3.01 and GraphPad Prism 5.00 (GraphPad Software Inc., California, USA) were used for data analysis.

## 3. Results

### 3.1. Clinical Characteristics of Patients

Thirty women were included in the study divided into normal pregnancies (*n* = 15) and preeclampsia (*n* = 15). Compatible with diagnostic criteria, women with preeclampsia exhibited higher systolic and diastolic blood pressure than normal pregnancy (Table 1). Gestational age at delivery, newborn weight, and height were lower in preeclampsia than normal pregnancy. 27% of pre-eclamptic women had small for gestational age babies. Despite the trend to lower placental weight in preeclampsia, it was not statistically significant compared to controls (Table 1).

### 3.2. Functional Characterization of A_2B_AR

**Figure 1:**
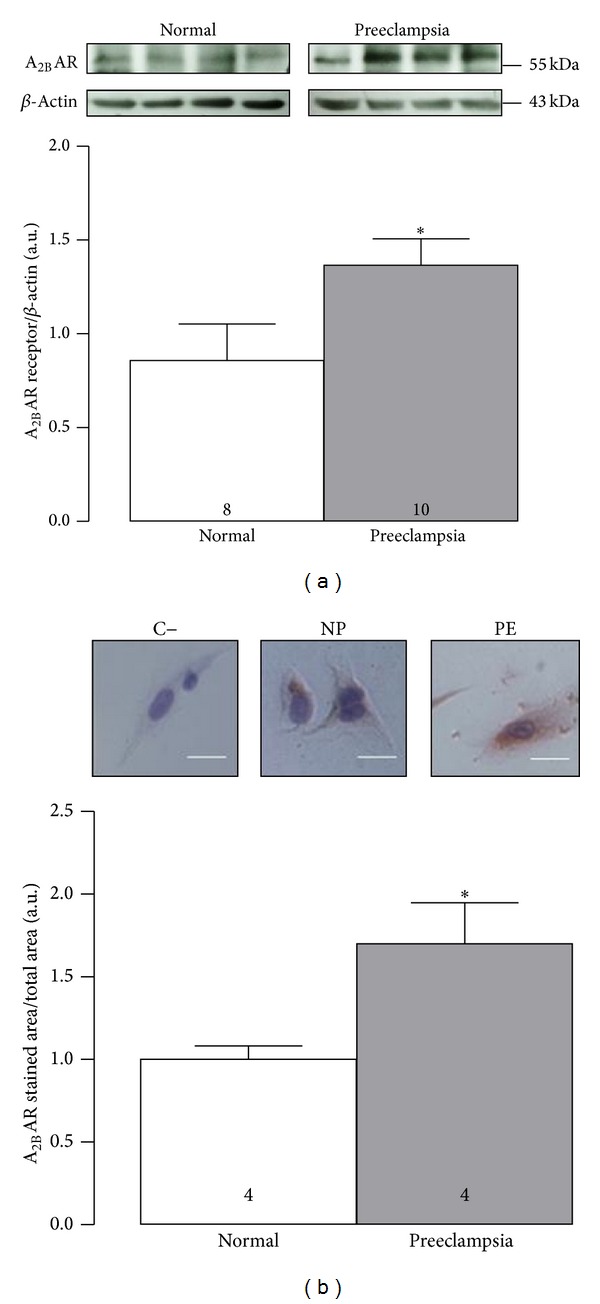
Protein levels of A_2B_AR in both normal and preeclamptic cells. (a) Cells derived from normal (NP) or preeclamptic pregnancies (PE) were used for estimating protein abundance of A_2B_AR by western blot. Upper panel shows representative images of A_2B_AR protein (55 kDa) and *β*-actin (43 kDa). Bottom panel shows densitometry of A_2B_AR/*β*-actin ratio. (b) Upper panel shows representative images of immunocytochemistry for A_2B_AR. Bottom panel included the ratio of the digital analysis of the stained area by A_2B_AR divided by total area as described in Section 2. Line in the photos represents 5 *μ*M. *P < 0.05 versus respective value in normal pregnancy. C− is negative control without primary antibody. Values are mean ± SEM. Respective *n* is indicated in each bar. All experiments were performed in duplicate.

**Figure 2:**
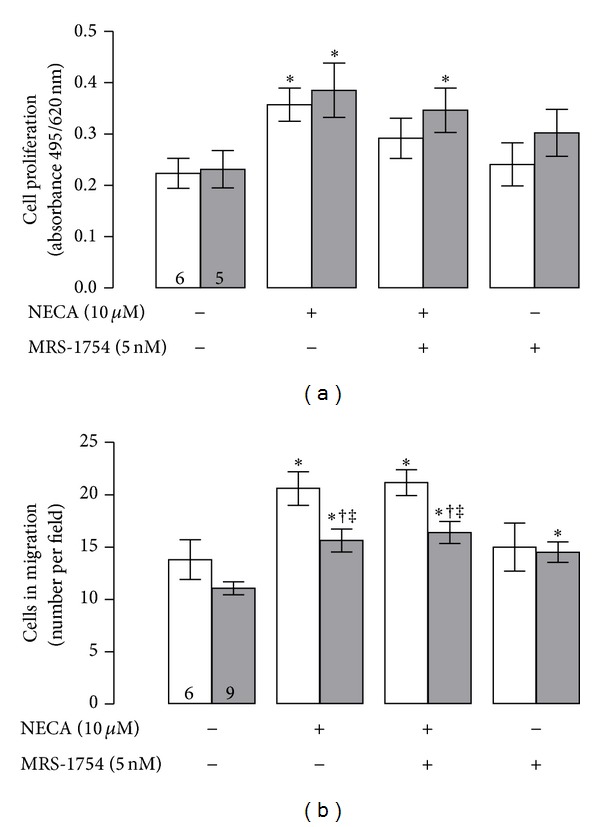
Cell proliferation and migration induced by A_2B_AR. HUVECs were isolated from normal (white bars) and preeclamptic pregnancies (grey bars) and used for (a) analysis of cell proliferation by MTS assay in presence (+) or absence (−) of NECA (10 *μ*M, 24 h) and/or MRS-1754 (5 nM, 24 h) or (b) cell migration as described in (a). *P < 0.05 versus basal condition in normal pregnancy. ^†^ *P* < 0.05 versus basal condition in preeclampsia. ^‡^ *P* < 0.05 versus respective value in normal pregnancy. Values are mean ± SEM. Values in respective control column indicate *n*. All experiments were performed in duplicate.

Western blot and immunocytology showed significantly higher (1.6 and 1.7, resp.) A_2B_AR protein levels in HUVEC from preeclampsia than those from normal pregnancy (Figure 1). In the presence of NECA (10 *μ*M, 24 h), it was observed a significant elevation in both proliferation (Figure 2(a)) and migration (Figure 2(b)) of HUVEC from normal and preeclamptic pregnancies. Moreover, NECA-induced cell proliferation was not observed when cells derived from normal pregnancies were coincubated with MRS-1754. However, in cells from preeclampsia, coincubation with NECA and MRS-1754 exhibited similar response to NECA alone. In addition, MRS-1754 did not affect cell proliferation in normal or pre-eclamptic pregnancies.

**Figure 3:**
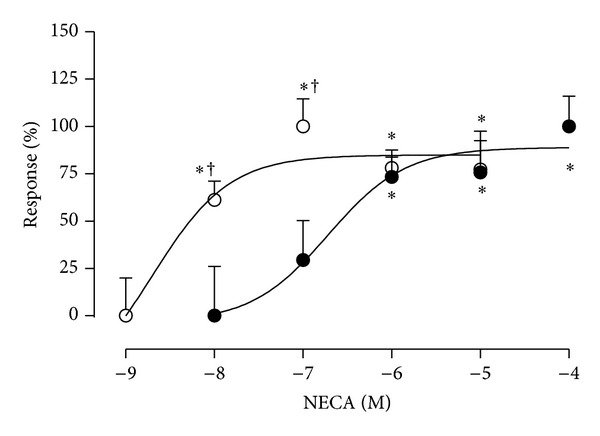
Cell proliferation induced by NECA in a concentration-response curve. HUVEC was isolated from normal (open circles, *n* = 6) and preeclamptic pregnancies (closed circles, *n* = 3) and used for cell proliferation in presence of NECA (10^−9^ to 10^−4^ M, 24 h). *P < 0.05 versus respective low NECA concentration. Values are mean ± SEM. All experiments were performed in duplicate.

The effect of NECA on cell proliferation was also tested in a concentration-response curve in both normal and preeclamptic derived cells (Figure 3). The calculated logarithmic EC_50_ (LogEC_50_) for NECA was significantly higher (−7.7 ± 0.5 and −9.6 ± 0.7 M, resp.; *P* < 0.05) in preeclampsia compared to normal pregnancy.

Regarding cell migration, despite the stimulatory effect observed after NECA incubation was similar in both normal and preeclamptic cells compared to its respective basal condition (1.5- and 1.4-fold, resp.), it was observed that cells from preeclampsia do not reach similar response compared to normal pregnancy (Figure 2(b)). In fact, migratory response was 29 ± 3% less in cells from preeclampsia compared to normal pregnancy. NECA-mediated cell migration was not affected by MRS-1754 co-incubation in cell from normal or pre-eclamptic pregnancies. Yet, cells from preeclampsia exposed to MRS-1754 alone exhibit a significant increase (1.3-fold) in cell migration compared to its basal condition without any treatment, whereas no effect was observed in cells from normal pregnancy incubated with this antagonist.

### 3.3. A_2B_AR Stimulation and NO

**Figure 4:**
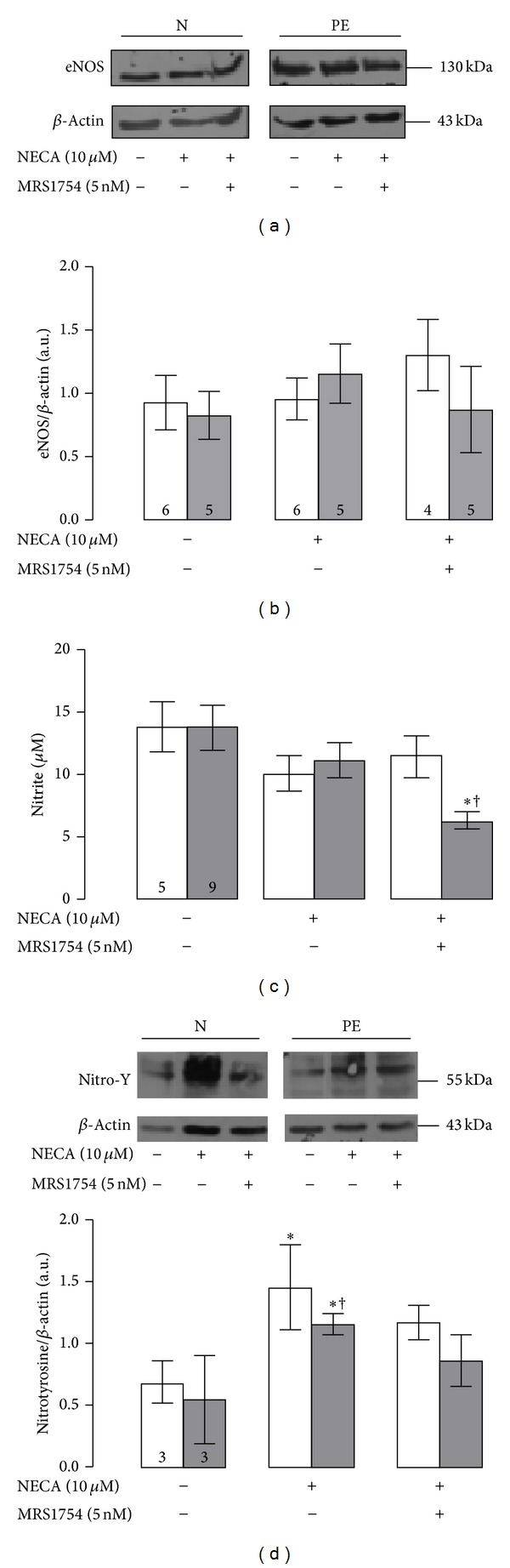
Stimulation of A_2B_AR generates nitrotyrosine formation. (a) Representative images of western blots for endothelial nitric oxide synthase (eNOS, 130 kDa) and *β*-actin (43 kDa) in absence (−) or presence (+) of NECA (10 *μ*M) and/or MRS-1754 (5 nM) during 12 hours in cells from normal (N) or preeclamptic pregnancies (PE). (b) Densitometry of the eNOS/*β*-actin ratio as showed in (a). (c) Nitrite concentrations in homogenate of cell after incubation (30 min) with NECA (10 *μ*M) and/or MRS-1754 (5 nM). (d) Upper panel, representative images of western blots for nitration of tyrosine residues (Nitro-Y) in protein(s) at 55 kDa and *β*-actin (43 kDa). Bottom panel presents the densitometry of the nitrotyrosine/*β*-actin ratio in the analyzed groups as showed in the upper panel. In (c) and (d), *P < 0.05 and ^†^ *P* < 0.05 versus value in basal condition (i.e., without any treatment) of normal pregnancy or preeclampsia, respectively. Values are mean ± SEM. Values in respective column indicate *n*. All experiments were performed in duplicate.

There were no differences in the protein abundance of endothelial nitric oxide synthase (eNOS) between preeclampsia and normal pregnancies (Figures 4(a) and 4(b)). Moreover, neither NECA (10 *μ*M) nor MRS-1754 incubation (5 nM, 12 h) changed the protein abundance of eNOS in either preeclampsia or normal pregnancy.

Nevertheless, during short incubations (30 min), NECA induce nonstatistically significant reduction in the nitrite levels measured in culture medium (Figure 4(c)), as well as a significant elevation in the intracellular nitrotyrosine formation (Figure 4(d)) in HUVEC from both preeclampsia and normal pregnancy. Moreover, coincubation of NECA + MRS-1754 was associated with reduction in the nitrite level only in preeclamptic cells. Furthermore, NECA-mediated nitrotyrosine formation observed in cells from normal or pre-eclamptic pregnancies was not blocked by MRS-1754.

**Figure 5:**
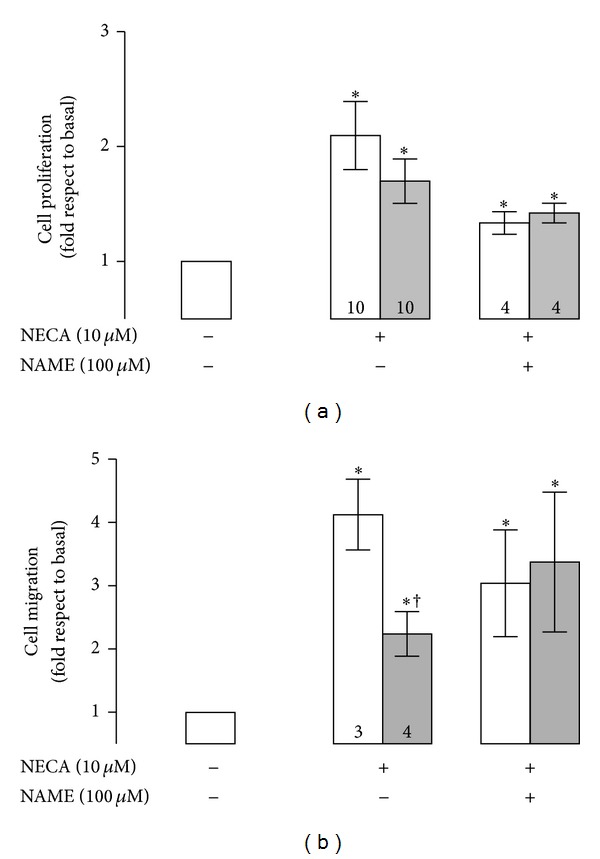
Effects of NOS inhibitor on NECA-mediated cell proliferation/migration. (a) Cell proliferation in presence (+) or absence (−) of NECA (10 *μ*M) and/or L-NAME (100 *μ*M) during 24 hours in normal (white bars) or preeclamptic pregnancies (grey bars). (b) Cell migration as in (a). *P < 0.05 versus respective value in basal conditions. ^†^ *P* < 0.05 versus respective value in normal pregnancy. Values are expressed in fold of change considering respective basal condition (i.e., without any treatment) in normal or preeclamptic cells. Values in mean ± SEM. Values in respective control column indicate *n*. All experiments were performed in duplicate.

In addition, increase in cell proliferation induced by NECA in normal or pre-eclamptic pregnancies (1.9 and 1.7 fold, resp.) (Figure 5(a)) was partially reduced by coincubation with L-NAME (100 *μ*M). Furthermore, when the percentage of reduction mediated by L-NAME coincubation was calculated, it was observed that normal pregnancy derived cells exhibited a higher percentage (38 ± 2%) than preeclampsia (18 ± 1%) (*χ* ^2^ = 8.9, *P* < 0.05). Nevertheless, combination of L-NAME and MRS-1754 reduced in 43 ± 1% and 29 ± 1% the NECA-mediated augmentation in cell proliferation in normal and preeclamptic HUVEC, respectively (data not shown). On the other hand, L-NAME induces a partial reduction (27 ± 4%) in the stimulatory effect of NECA on cell migration, whereas no affect was observed in preeclampsia (Figure 5(b)).

### 3.4. A_2B_AR Stimulation and VEGF

**Figure 6:**
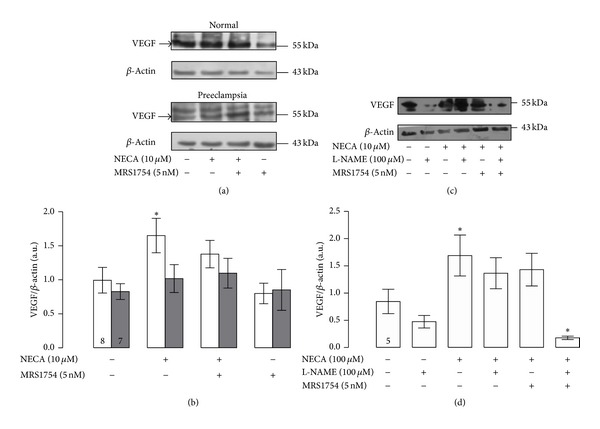
Effect of A_2B_AR stimulation in the VEGF protein level. (a) Representative images of western blot for VEGF (55 kDa) and *β*-actin (43 kDa) in HUVEC from normal or preeclamptic pregnancies incubated in absence (−) or presence of NECA (10 *μ*M) and/or MRS-1754 (5 nM) during 12 hours. (b) Densitometry of the VEGF/*β*-actin ratio as showed in (a). (c) Representative images of VEGF (55 kDa) and *β*-actin (43 kDa) in HUVEC from normal pregnancies treated with NECA (10 *μ*M) and/or MRS-1754 (5 nM) and/or L-NAME (100 *μ*M) during 12 hours. (d) Densitometry of the VEGF/*β*-actin ratio as showed in (c). *P < 0.05 versus basal condition in normal pregnancy. Values are mean ± SEM. Values in respective control column indicate *n*. All experiments were performed in duplicate.

There were no significant differences in the protein level of VEGF in HUVEC derived from normal and preeclampsia. After stimulation with NECA (10 *μ*M, 12 h), cells from normal pregnancies exhibited an increase (1.7-fold) in the VEGF protein abundance compared to corresponding controls without any treatment. The latter effect of NECA was not observed in cells coincubated with MRS-1754 (5 nM). Contrarily, neither NECA nor MRS-1754 alone or in combination affected the VEGF protein level in preeclampsia (Figures 6(a) and 6(b)).

### 3.5. NO and VEGF Expression

The stimulatory effect of NECA (10 *μ*M, 12 h) on VEGF protein levels was partially blocked by L-NAME or MRS-1754 (Figures 6(c) and 6(d)). Interestingly, the reductive effect of these last two inhibitors upon NECA effect was more than additive since cell coincubated with L-NAME, MRS-1754, and NECA exhibited reduced VEGF levels even below control condition without any treatment.

## 4. Discussion

Results presented in this work describe that HUVEC from preeclampsia exhibits elevated protein level of A_2B_AR compared to normal pregnancy. However, functionality of this receptor may be reduced in preeclampsia. This is supported by results showing a shift in the concentration-response curve to NECA during cell proliferation, where logEC_50_ is higher in preeclampsia than normotensive pregnancies. In addition, a reduced migratory response to NECA was seen in preeclampsia compared to normal pregnancy; and contrary to cell from normal pregnancy, in pre-eclampsia the expression of VEGF associated with A_2B_AR stimulation was absent. Results from coincubation of agonist and antagonists suggest that activation of A_2B_AR triggers intracellular pathways involving protein nitration, which may participate in cell proliferation/migration and VEGF protein expression in HUVEC from normal pregnancies. However, in preeclampsia only cell proliferation seems to be associated with protein nitration induced by A_2B_AR. In conclusion, HUVECs from preeclampsia exhibit elevated protein level of A_2B_AR. Moreover, tyrosine nitration and VEGF protein expression mediated by A_2B_ are associated with cell proliferation/migration in normal cells, but this cell signaling is impaired in preeclampsia.

### 4.1. Clinical Context

Preeclamptic placenta exhibits reduced fetoplacental blood flow [4], generated by placental under perfusion [2, 3] associated with limited placental invasion into maternal spiral arteries [33] and impaired remodeling process of those vessels [34]. Furthermore, hemodynamic changes generated by those alterations in the maternal-placental interphase may generate a turbulent blood flow that hits the placenta and leads to impaired placental villi architecture [35], then compromising the fetoplacental circulation in preeclampsia [4]. All these alterations have been associated with generation of oxidative and nitrative stress within the placenta [36, 37], leading to endothelial dysfunction in the fetoplacental circulation [11–14]. Others and we believe that part of this endothelial dysfunction present in the preeclamptic placenta may include alteration in the angiogenesis process [3, 4, 14, 29, 38], which in turn could explain the elevated risk for short and long term complications observed in children exposed to preeclampsia intrauterine. Regarding short-term complication associated to preeclampsia, clinical data presented in this study, showing that preeclamptic women exhibited high blood pressure (mean value 161/102 mmHg), associated with reduced gestational age and 4 of 15 preeclamptic women had a baby small for gestation age. Although we did not analyze the data according to the severity of the disease or gestation age of presentation, our study describes major differences in the A_2B_AR/NO/VEGF signaling pathway when cells from preeclampsia are compared with those derived from normotensive pregnancies. However, the impact of confounding variables including degree of severity of the disease, gestational age at onset, treatment received, and children sex should be addressed in future studies.

### 4.2. Adenosine and HUVEC Proliferation/Migration

Adenosine promoted angiogenesis. The underling mechanisms are under investigation and include direct and indirect actions on several different cell types and practically all adenosine receptors may be involved [23, 39]. Focusing on endothelial cells, either A_2A_AR or A_2B_AR has been shown to mediate the proliferative actions of adenosine in human retinal microvascular endothelial cells [15, 40], HUVEC [22, 29], or porcine coronary artery and rat aortic endothelial cells [28], while it remains still unclear if A_1_AR and A_3_AR are functionally expressed, and what role, if any, they play in endothelial cells.

Particularly, expression of A_2B_AR in HUVEC and its participation in angiogenesis have been well characterized in the literature [22, 27, 39, 41–43], which is also supported by our results. Moreover, functional presence of A_2B_AR leading to activation of NO/VEGF signaling pathway in cells derived from normal pregnancy is suggested because the NECA-mediated augmentation in cell proliferation, nitrotyrosine formation, and VEGF protein abundance is inhibited by MRS-1754 coincubation. These results agree with previous observations regarding induction of NO production after A_2A_AR/A_2B_AR stimulation in porcine carotid artery endothelial cells (PCAEC) [44], human iliac arterial endothelial cells (HIAEC) [45], and HUVEC [27, 29, 46, 47], as well as with others indicating adenosine mediated augmentation in the VEGF expression in many endothelial cells [29, 40, 41, 48–53].

Moreover, our results in HUVEC derived from normal pregnancy suggest that A_2B_AR-mediated activation of NO synthesis would not be the unique pathway involved in the cell proliferation/migration in HUVEC. This is because NECA-mediated increase in cell proliferation/migration and VEGF protein abundance was only partially reverted by the NOS inhibitor, L-NAME, whereas coincubation with both inhibitors MRS-1754 (A_2B_AR antagonist) and L-NAME has additive effects upon these two NECA-mediated effects. It is unknown how NO/protein nitration may control cell proliferation and VEGF expression in HUVEC. However, potential mechanism might include activation of hypoxia inducible factor mediated by nitration [54].

In our study, we used the unspecific inhibitor of NOS, L-NAME, in a concentration (100 *μ*M) that should be inhibiting all the isoforms of NOS according to the estimated value of K*i* (neuronal NOS (nNOS, 15 nM), eNOS (39 nM), and inducible NOS (iNOS, 65 *μ*M)). Although our cell model expresses constitutively eNOS, we could not roll out the participation of other isoforms in our experimental data. Another limitation regarding importance of NO in our study is the use of nitrite measurements and nitrotyrosine formation as indirect markers for NO synthesis. Although both techniques have several advantages and disadvantages for estimating NO synthesis, it is clear that more direct measurement is required in order to elucidate participation of NO in the A_2B_AR signaling pathway. Therefore, future studies should consider the use of more specific inhibitors for eNOS, or molecular biology techniques for suppressing or overexpressing eNOS.

### 4.3. Preeclampsia, Adenosine, and HUVEC Proliferation/Migration

In the preeclampsia field, elevated level of plasma adenosine has been reported in the fetoplacental circulation [7, 19, 20], which reaches the micromolar range in umbilical vein; therefore it is feasible that under these conditions all adenosine receptors would be stimulated [18, 55]. Moreover, elevation of adenosine in the fetal circulation of preeclamptic pregnancies seems to be a phenomenon depending on the severity of the disease, since only children with alteration in the Doppler velocimetry of umbilical artery exhibit elevation in the adenosine level compared to normal pregnancy [20]. Causes and consequences of elevated extracellular adenosine concentration in preeclampsia are unclear.

We have previously documented that HUVECs isolated from late-onset preeclampsia exhibit high cell proliferation/migration, while in early onset preeclampsia these parameters were reduced in relation to women with normal pregnancy [29]. Interestingly, activation of A_2A_AR was heterogeneous between late-onset preeclampsia and early onset preeclampsia. Thus, whereas late-onset preeclampsia was associated with a “basal” activation of the adenosine/NO/VEGF pathway, early onset preeclampsia exhibited a downregulation of this particularly via [29], suggesting that changes in cell proliferation/migration observed between late-onset preeclampsia and early onset preeclampsia may be explained by changes in the A_2A_AR/NO/VEGF signaling pathway activation.

In the actual report, although we did not classify the women in late-onset or early onset preeclampsia, due to sample size, most of the preeclamptic women belong to group with early onset preeclampsia, as indicated by reduced gestational age at delivery. In addition, in order to avoid potential “basal” effect of high extracellular level of adenosine, we have performed all experiments in presence of adenosine deaminase. Under these experimental conditions, an increased protein abundance of A_2B_AR associated with reduction in its function is present in HUVEC from preeclampsia. These results allow speculating the following. (1) Elevation in the A_2B_AR observed in preeclampsia may be associated with activation of hypoxia inducible factor 1 alpha (HIF-1*α*) [56], since the A_2B_AR promoter contains a functional binding site for HIF-1 *α* [57], which promotes A_2B_AR expression in HUVEC [22]. (2) The reduced activation of A_2B_AR observed in preeclampsia may be related to reduced availability of the receptor in the cell membrane. Indirect evidences for this hypothesis are elevated logEC_50_ for NECA-mediated proliferation observed in HUVEC from preeclampsia compared to normal pregnancy. Clearly, more studies are required to elucidate the mechanisms linked with A_2B_AR expression and activation in preeclampsia.

Result in this study suggests that A_2B_AR/NO/VEGF pathway observed in normal pregnancy would be dysfunctional in HUVEC from preeclampsia. This idea is supported by the fact that although activation of A_2B_AR is associated with nitration and cell proliferation, it was not related to VEGF protein expression. In this regard, Feoktistov and colleagues [22] have reported that hypoxia-mediated upregulation of A_2B_AR in HUVEC has a functional impact, since only under this condition the A_2B_AR was coupled to upregulation of VEGF. Our results partially agree with this concept, with respect to upregulation of A_2B_AR in preeclampsia, a condition characterized by placental under perfusion [2, 3] and HIF-1 *α* activation [56]. Contrarily, we did not find an A_2B_AR-mediated VEGF expression in preeclampsia, whereas it was evidenced in cell from normal pregnancy. Apparent discrepancy may be related to experimental condition, since in the Feokstitov's report [22] they use a HUVEC cell line, rather than primary culture as we reported; and hypoxia was defined as 4.6% oxygen, which is considered normoxia for primary culture of HUVEC [58].

There are some intriguing results in our study that we would like to discuss. For instance, A_2B_AR activation might not be associated with cell migration in either normal pregnancy or preeclampsia, because MRS-1754 was unable to block the stimulatory effect of NECA upon cell migration. Although we did not analyze the participation of other adenosine receptors in this particular study, it has been described previously that activation of A_2A_AR increases cell migration in HUVEC from either normal or preeclamptic pregnancies [29]. Then, A_2A_AR, rather than A_2B_AR, may be involved in the cell migration during normal pregnancy and preeclampsia. Another intriguing result is the reduction in the nitrite levels (i.e., NO metabolites) in the culture medium of preeclamptic cells induced by coincubation with NECA and MRS-1754 (Figure 4(c)). As stated above, we could not roll out the participation of other adenosine receptors (except A_2B_AR) in this response.

## 5. Conclusion

HUVECs from normal pregnancies exhibit a functional presence of A_2B_AR, whose activation is associated with cell proliferation, mediated at least partially, via intracellular protein nitration and VEGF synthesis. On the other hand, cells from preeclampsia are characterized by upregulation in the A_2B_AR protein expression, but its activity is diminished and might not be involved in the control of VEGF expression.

## Figures and Tables

**Table 1:** Characteristics of the included women.

	Normal	Preeclampsia
	(*n* = 15)	(*n* = 15)
Age (years)	25.1 ± 1.6	26.5 ± 1.7
BMI at delivery (kg/m^2^)	31.4 ± 1.3	33.5 ± 1.4
GA at delivery (wk)	39.4 ± 0.3	37.4 ± 0.3*
SBP (mmHg)	115.2 ± 1.5	161.9 ± 4.8*
DBP (mmHg)	72.7 ± 1.7	102.1 ± 2.3*
Proteinuria, g/24 h	0	2.2 ± 1.0*
Newborn		
Male/female	7/8	7/8
Weight (g)	3333 ± 111.3	2726 ± 178.8*
Height (cm)	48.8 ± 0.4	46.3 ± 0.7*
SGA, *n* (%)	0	4 (27)*
Cephalic perimeter (cm)	34.1 ± 0.2	33.3 ± 0.6
Placenta		
Weight (g)	542.7 ± 33.5	490.0 ± 34.6
Area (m^2^)	3.3 ± 0.3	2.8 ± 0.2
NBW/PlW (g/g)	6.3 ± 0.3	5.7 ± 0.2

BMI: body mass index; SBP: systolic blood pressure; DBP: diastolic blood pressure; SGA: small for gestational age (<10th percentile according to gestational age); NBW: newborn weight; GA: gestational age; PlW: placental weight. *P < 0.05 versus normal pregnancy. Values are mean ± SEM.

## Abbreviations

NO: Nitric oxide
NOS: Nitric oxide synthase
VEGF: Vascular endothelial growth factor
A_2A_AR: A_2A_ adenosine receptor
A_2B_AR: A_2B_ adenosine receptor
L-NAME: NG-nitro-L-arginine methyl ester
NO: Nitric oxide
VEGF: Vascular endothelial growth factor
HUVECs: Human umbilical vein endothelial cells
hPMEC: Human placental microvascular endothelial cells.
